# In Vitro Assessment of *Balanites aegyptiaca* Fruit Methanolic Extract on the Adult *Toxocara canis*

**Published:** 2018

**Authors:** Hatem SHALABY, Amira EL NAMAKY, Omnia KANDIL, Noha HASSAN

**Affiliations:** Dept. of Parasitology and Animal Diseases, National Research Center, Giza, Egypt

**Keywords:** *Toxocara canis*, *Balanites aegyptiaca* fruits, In vitro effect, Cuticle, Scanning electron microscopy

## Abstract

**Background:**

The effectiveness of anthelmintics may diminish within approximately 10 yr of use relying on several factors such as anthelmintic resistance. This study aimed to assess in vitro effect of *Balanites aegyptiaca* fruits extract on the cuticle of adult worm of *Toxocara canis* as naturally alternative therapy.

**Methods:**

*B. aegyptiaca* fruits were procured from the local markets in Aswan, Upper Egypt and authenticated at the Herbarium of National Research Centre. The effect of methanolic extract of *B. aegyptiaca* fruits on adult *T. canis* after 24 and 48 h incubating the parasites in Ringer solution containing 240 μg/ml *Balanites* extract was determined by scanning electron microscopy.

**Results:**

The main changes induced by treatment with the tested extract were wrinkled cuticular surface and deformed sensory papillae. This cuticular distortion would undoubtedly disrupt its protective function and might be enough to expel *Toxocara* worms from dog’s intestine.

**Conclusion:**

The use of this plant offers a chance for new nematocidal agent, which is economical alternative for the more expensive anthelmintics.

## Introduction

*Toxocara canis* is probably the most common intestinal helminth of dogs worldwide and has, because of its zoonotic significance, important public health consequences (1). The puppies and young dogs are more susceptible to *Toxocara* infection than adult animals. Infection can be acquired either orally by ingestion of embryonated *Toxocara* eggs from contaminated environment or transplacentally from infected mother. Toxocariasis is the clinical term applied to infection in dogs. Its controlling by using anthelmintics drugs improves the dog’s health and minimizes the hazard of infection in humans (2). Besides, the effectiveness of anthelmintics may diminish within approximately 10 yr of use relying on several factors such as anthelmintic resistance (3). Therefore, the use of medicinal plants as alternative therapy should be one of the main considerations.

The use of medicinal herbs has increased over the past few years “back to nature” instead of using synthetic drugs which may have disadvantages that may be more serious than the disease itself. *Balanites aegyptiaca* (*B. aegyptiaca*) is one of the most common wild plant species distributed in African and known by its many uses for medicinal purposes (4). The fruit, which is safe to eat, gives a worthy oil and also has saponins which are fatal to definite invertebrates and consequently of importance in getting rid of the carriers of guinea-worm (4) and schistosomiasis (5). Its methanolic extract showed destructive effects on cuticle of biologically related nematode *Toxocara vitulorum* (6) and tegument of rumen flukes *Paramphistomum microbothrium* (7). In Egypt, the prevalence of *T. canis* in stray dogs examined was 56% (8), with increasing the possibility of dissemination of *T. canis* eggs out of the environment and risk-infecting *T. canis* larvae in human.

The purpose of this study was to determine in vitro activity of *B. aegyptiaca* fruits methanolic extract on the cuticle of adult worm of *T. canis* which is vital for the protective function.

## Materials and Methods

### Preparation of methanolic extract of *B. aegyptiaca* fruits

*B. aegyptiaca* fruits were procured from the local markets in Aswan, Upper Egypt and authenticated at the Herbarium of National Research Centre. Their methanolic extract was obtained (9). Briefly, the kernels of fruits were removed and the remaining mesocarps (1 kg) were soaked in methanol 90% for 48 h, and then subjected to extraction in Soxhlet apparatus till complete exhaustion. The methanolic extract was concentrated under reduced pressure on a rotatory evaporator and then dissolved in a vehicle mixture of liquid paraffin and Tween 80 (v/v) to obtain a 10% liquid extract.

### Recovery and in vitro treatment of adult *T. canis*

Adult *T. canis* were collected from the intestines of naturally infected stray dogs that were killed by Egyptian police. After recovery, the worms were identified according to the method (10) and washed in body-warm Ringer solution. Under sterile conditions in a laminar flow cabinet, whole worms were transferred to Ringer solution containing *B. aegyptiaca* extract at a concentration of 240 μg/ml. Stock solution of methanolic extract at 10 mg/ml was prepared with a mixture of liquid paraffin and Tween 80 (v/v) for immediate use. Then, the whole worms were incubated for 24 and 48 h at 37 °C in an atmosphere of 5% CO_2_. The concentration was chosen on the basis of concentrations used in vitro with experiments involving *T. vitulorum* (6) and *P. microbothrium* (7). Solvent control worms were incubated for 24 and 48 h in Ringer solution containing 0.2% (v/v) mixture of liquid paraffin and Tween 80. Normal control worms were fixed immediately following the initial washing. Five worms were examined for each time period. The activity of worms was monitored at 24 and 48 h incubation by observation and if necessary by mechanical excitation with forceps.

### Scanning electron microscopy (SEM)

Following incubation with 240 μg/ml *B. aegyptiaca* extract, the anterior end of adult worms of *T. canis* was fixed intact for 12 h in a 3:1 mixture of 4% (w/v) glutaraldehyde in 0.12 M Millonig’s buffer, pH 7.4 and 1% aqueous osmium tetroxide. After this, the specimens were processed for SEM following a method previously reported (6).

## Results

The control *T. canis* adult worms demonstrated no loss of action amid the entire time of incubation (48 h) and responded much more sensitively to changes in the surrounding conditions than the treated worms. Yet, no paralysis was observed in the latter worms.

### Scanning electron microscopy of the anterior end of normal and control worms

**Fig. 1:**
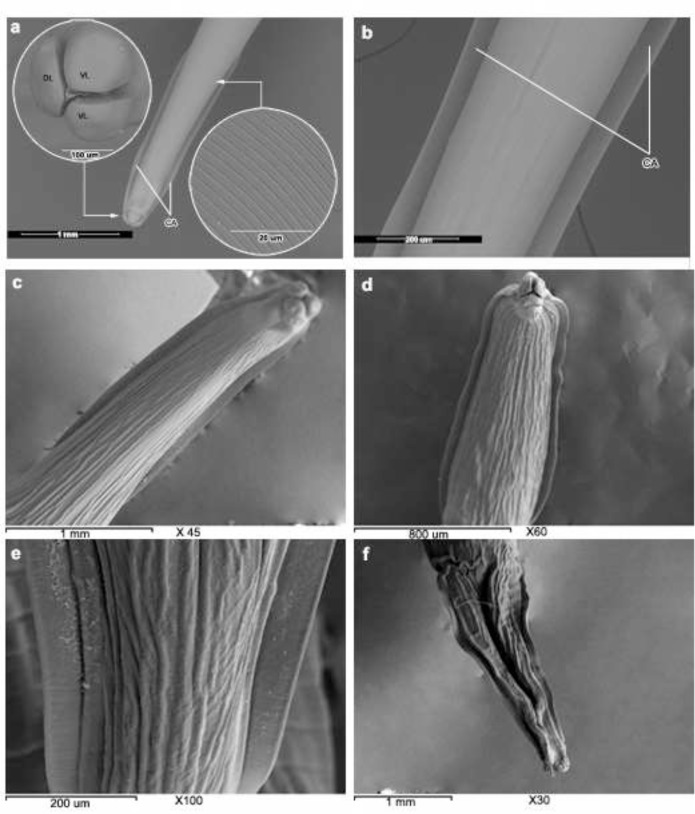
Scanning electron micrographs (SEMs) of adult *Toxocara canis*. **(a, b)** Normal fresh worms. SEMs of the anterior end show three prominent lips, one dorsal (DL) and two ventro-laterals (VL), and cervical alae (CA) which are present as lateral lance shaped ridges. The cuticle is transversally striated (*inset*). **(c- e)** Following 24 h incubation with *Balanites aegyptiaca* extract. SEMs of the anterior end show wrinkled cuticular surface and less pronounced transverse striation with longitudinal folds. **(f)** Following 48 h incubation with *Balanites aegyptiaca* extract. SEM of the anterior end show severe wrinkling and corrugation of the cuticular surface

The anterior end and its structure of untreated adult *T. canis* had been previously mentioned. Briefly, it showed three prominent lips, one dorsal and two ventro-laterals. The dorsal lip exhibited two large external papillae associated with a pair of large amphids. Each subventral lip exhibited one large and one small papilla; alongside them, a pair of amphids was observed. Each lip had a dentigerous ridge and two small inner labial papillae similarly as distinct pits near its outer margin. The cervical alae were present as lateral lance-shaped ridges. The cuticle was transversally striated; sets of striations were separated by deep groove-like annulations giving the cuticle a divided appearance. These portions had a smooth surface (Fig. 1a, b). No significant differences were observed between normal and control worms incubated for 48 h in medium with 0.2% solvent addition.

### Effects of methanolic extract of B. *aegyptiaca* fruits on the cuticle of adult worms

After 24 h incubation with *B. aegyptiaca* extract, the cuticle of the anterior end showed wrinkled cuticular surface; so that the transverse striation became less pronounced and lost their normal aspect showing longitudinal folds (Fig.1c, d, e). By 48 h incubation, the wrinkling of the cuticle was more pronounced, with lips showed wrinkled cuticular surface and deformed sensory papillae (Fig.1f).

## Discussion

There is a particular interest in studies about in vitro activity against the adult as well as the larval stages some of the parasitic helminths. Appropriate anti-helminthic effects demonstrated by its ability for rapid preparation, higher availability, stronger effects at low concentrations, high efficacy in shorter time after exposure, and less toxic effects (11, 12).

During the entire time of worm incubation (48 h), in the present study, the controls remained active and responded to changes, in the surrounding condition, much more sensitively than the treated *Toxocara* worms. However, none of the latter worms showed paralysis. This depression of activity might be enough to dislodge *Toxocara* worms from dog’s intestine, as had been demonstrated for other dog’s anthelmintics (1,13). This study was the first one investigating in vitro the efficacy of *B. aegyptiaca* fruits extract against *T. canis* adult worms; the cuticular alterations were assessed by using scanning electron microscopy. The main changes induced by treatment with the tested extract were wrinkled cuticular surface and deformed sensory papillae. This cuticular distortion would undoubtedly disrupt its protective function. These results appeared to be in line with previous in vitro studies that showed similar surface distortion of biologically related nematode, *T. vitulorum* (6) and specimens of trematode, *Paramphistomum microbothrium* (7) following their exposure to *B. aegyptiaca* fruits extract. Moreover, the cuticular surface alterations induced by *B. aegyptiaca*, in the present study, had also been observed following treatment with artemether, obtained from the leaves of qinghao as well as albendazole, which was 100% effective against adult *T. canis* (1).

Surface deformity caused by either synthetic or natural anthelmintics appeared to be as a common feature of drug-treated parasites and might be attributed to passive diffusion of anthelmintics through the body surface (14). Indeed, nematocidal drugs such as albendazole and its related compound enter the parasite body through simple diffusion and cause distortion of the nematode body surface (6, 15). *B. aegyptiaca* fruits methanolic extract was suggested to have a wide range of anthelmintics activity, and its effect might be attributed to its saponin constituents (16). Balanitin-7, a steroidal saponin isolated from *B. aegyptiaca*, was tested against *Caenorhabditis elegans* adult worm’s viability (17). Balanitin-7 showed high potential anthelmintic activity and was more potent than some well-known anthelmintics such as pyrantel and piperazine. The surface active and highly cytotoxic properties of saponins to which balantins belong were what distinguished these compounds from other glycosides (16). This might be explained the body wall destructive effects of *Balanites* extract on *T. canis* adult worms.

## Conclusion

The methanolic extract of *B. aegyptiaca* fruits has a potent effect on the cuticle of adult *T. canis* in vitro. The use of this plant offers a chance for new nematocidal agent, which is economical alternative for the more expensive anthelmintics. However, its effectiveness in killing the parasite in vivo needs to be established.
